# Free Volume and Permeability of Mixed Matrix Membranes Made from a Terbutil-*M*-terphenyl Polyamide and a Porous Polymer Network

**DOI:** 10.3390/polym14153176

**Published:** 2022-08-03

**Authors:** Cenit Soto, Javier Carmona, Benny D. Freeman, Laura Palacio, Alfonso González-Ortega, Pedro Prádanos, Ángel E. Lozano, Antonio Hernandez

**Affiliations:** 1 Surfaces and Porous Materials (SMAP), Associated Research Unit to CSIC, University of Valladolid, Facultad de Ciencias, Paseo Belén 7, E-47011 Valladolid, Spain; marveliacenit.soto@uva.es (C.S.); fcojavier.carmona@uva.es (J.C.); laura.palacio@uva.es (L.P.); pradanos@termo.uva.es (P.P.); lozano@ictp.csic.es (Á.E.L.); 2 Institute of Sustainable Processes (ISP), Dr. Mergelina s/n, 47011 Valladolid, Spain; 3 McKetta Department of Chemical Engineering, Texas Materials Institute, The University of Texas at Austin, Austin, TX 78712, USA; freeman@che.utexas.edu; 4 Department of Organic Chemistry, School of Sciences, University of Valladolid, Facultad de Ciencias, Paseo Belén 7, E-47011 Valladolid, Spain; alfonso.gonzalez.ortega@uva.es; 5 Institute for Polymer Science and Technology (ICTP-CSIC), Juan de la Cierva 3, 28006 Madrid, Spain; 6 IU CINQUIMA, University of Valladolid, Paseo Belén 5, E-47011 Valladolid, Spain

**Keywords:** hydrogen separation, mixed matrix membranes, porous polymer networks, thermal rearrangement

## Abstract

A set of thermally rearranged mixed matrix membranes (TR-MMMs) was manufactured and tested for gas separation. These membranes were obtained through the thermal treatment of a precursor MMM with a microporous polymer network and an o-hydroxypolyamide,(HPA) created through a reaction of 2,2-bis(3-amino-4-hydroxyphenyl)-hexafluoropropane (APAF) and 5′-terbutil-*m*-terfenilo-3,3″-dicarboxylic acid dichloride (tBTmCl). This HPA was blended with different percentages of a porous polymer network (PPN) filler, which produced gas separation MMMs with enhanced gas permeability but with decreased selectivity. The thermal treatment of these MMMs gave membranes with excellent gas separation properties that did not show the selectivity decreasing trend. It was observed that the use of the PPN load brought about a small decrease in the initial mass losses, which were lower for increasing PPN loads. Regarding the glass transition temperature, it was observed that the use of the filler translated to a slightly lower Tg value. When these MMMs and TR-MMMs were compared with the analogous materials created from the isomeric 5′-terbutil-*m*-terfenilo-4,4″-dicarboxylic acid dichloride (tBTpCl), the permeability was lower for that of tBTmCl, compared with the one from tBTpCl, although selectivity was quite similar. This fact could be attributed to a lower rigidity as roughly confirmed by the segmental length of the polymer chain as studied by WAXS. A model for FFV calculation was proposed and its predictions compared with those evaluated from density measurements assuming a matrix-filler interaction or ideal independence. It turns out that permeability as a function of FFV for TR-MMMs follows an interaction trend, while those not thermally treated follow the non-interaction trend until relatively high PPN loads were reached.

## 1. Introduction

The current energy emergency and global warming crisis require the development of new methods for a cost-efficient hydrogen purification [1], the removal of CO_2_ from natural gas or biogas [2,3], hydrocarbon separation [4], etc. Over the past years, polymeric membranes have become a widely implemented technology in a variety of industrial gas separation applications [2]. Polymeric membranes exhibit multiple advantages such as a low energy consumption, easy operation, small footprint and a low mechanical complexity [5]. However, the performance of polymeric membranes is still impacted by the trade-off between permeability and selectivity, represented by the “Robeson upper limit [6,7,8], their low chemical and thermal stability and plasticization at high CO_2_ pressures [9]”.

Thermally rearranged (TR) polymers with polybenzoxazole (PBO) moieties have emerged as a promising material in gas separation due to their outstanding performance in CO_2_ separation from gas energy vectors (i.e., biogas and biohydrogen), exceeding the upper limit of Robeson (2008) [10]. This exceptional performance is attributed to a bimodal “hourglass” pore architecture formed during structural rearrangement at elevated temperatures (350–450 °C) [10]. In this context, mixed matrix membranes (MMMs) have also emerged in recent years as an alternative to overcome the trade-off between permeability and selectivity. These hybrid materials benefit from the processability of polymers by adding organic/inorganic materials in order to form such hybrid materials [11,12].

In this context, a wide variety of filler materials has been used as fillers to manufacture these novel materials, including hyper-crosslinked polymers [13], metal-organic frameworks [14,15], zeolitic imidazolate frameworks [9,16], porous aromatic frameworks [17], porous polymer networks (PPNs) [18], etc. These fillers have been shown to enhance the gas transport properties of polymer matrixes. In addition, recent studies have also shown that the use of these materials as fillers can inhibit associated physical aging and the corresponding decrease in permeability [19,20,21].

Furthermore, the combination of TR-able polymers and microporous fillers can result in thermally rearranged mixed matrix membranes (TR-MMMs), which can improve permeability and selectivity over pure polymers and original MMMs [9,12,17,22]. These novel materials benefit from the properties of the TR-able materials formed through the conversion of *ortho*-hydroxyl-imides or *ortho*-hydroxyl-amides to benzoxazole groups at high temperatures. Despite the promising characteristics of TR-MMMs, the number of studies devoted to assessing their performance during gas separation is scarce [17,23,24].

This work aimed at manufacturing and testing novel polymeric gas separation membranes, MMMs and TR-MMMs, with enhanced gas transport properties for gas separation using PPNs synthesized using triptycene and trifluoroacetophenone as fillers, employing a SEAr methodology [25], with high thermal stability and a moderate surface area. The polymer matrix synthesis was based on the reaction between a diamine and a dichloride acid with 5′-tert-butyl-m-terphenyl (tBT) groups, where the reactive group was placed in the meta position (5′-terbutil-*m*-terphenyl-3,3″-dicarboxylic acid dichloride, tBTmCl). The *o*-hydroxy diamine used was 2,2-bis (3-amino-4-hydroxy phenyl)-hexafluoropropane (APAF). Excellent separation properties were recently obtained by following a similar synthetic route but with 5′-terbutil-*m*-terfenilo-4,4″-dicarboxylic acid dichloride (tBTpCl) with the acid chloride groups placed in the para position [26]. The combination of these two materials to fabricate MMMs, specifically the addition of PPNs in a polymeric matrix capable of producing benzoxazole groups, was carried out to improve the performance of the polymeric membranes. These resulting MMMs were subjected to a heat treatment (375 °C) to produce the TR-MMMs. The permeation mechanisms in the MMMs were analyzed here by using fractional free volume (FFV) evaluated both with and without interactions between the filler and polymeric matrix from the membrane densities. This research was conducted in order to elucidate if filler inclusions were surrounded by loose or tight shells, thus, increasing or decreasing permeability and selectivity as a function of the filler content of the MMMs.

## 2. Materials and Methods

Anhydrous dimethyl acetamide (DMAc, 99%), anhydrous pyridine (Py), dimethyl amino pyridine (DMAp), trimethylsilylchloride (TMSC, >98%), N, N dimethyl formamide (DMF), 3-methoxycarbonyl-phenylboronic acid and tetrahydrofuran (THF, 99%) were purchased from Sigma Aldrich (Sigma Aldrich, St. Louis, MO, USA) and used as received. NaOH (sodium hydroxide), sulfuric acid (H_2_SO_4_), Tetrakis(triphenylphosphine) palladium (0) (Pd(PPh_3_)_4_), potassium carbonate (K_2_CO_3_), hydrochloric acid and thionyl chloride (SOCl_2_) were obtained from Scharlau (Scharlab, Barcelona, Spain). The diacid 5′-tert-butyl-*m*-terphenyl-3,3″-dicarboxylic acid (tBTmDA) was synthesized following the procedure previously reported [27].

o-Hydroxydiamine 2,2-bis(3-amino-4-hydroxy phenyl)-hexafluoropropane (APAF) was purchased from Apollo Scientific (Apollo Scientific, Stockport, Cheshire, UK) and purified through sublimation at 220–225 °C before use. 4,4′-(hexafluoroisopropylidene) dianiline (6FpDA, Apollo Scientific, Stockport, Cheshire, UK) purified through sublimation at 220 °C and 5′-terbutil-*m*-terphenyl-3,3″-dichloride (tBTmCl) were employed as monomers to create the polyamide without o-hydroxy groups (PA). The synthesis of the corresponding HPA was carried out as described elsewhere [28].

The dichloride monomer (tBTmCl) was prepared from the corresponding diacid, as described below.

### 2.1. Synthesis of 3-Carboxy-phenylboronic Acid Results

**Figure 1:**
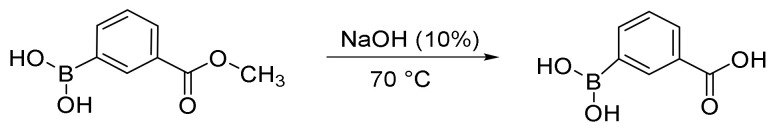
Synthesis of 3-carboxy-phenylboronic acid.

In a round-bottomed flask equipped with a reflux condenser, 15 g of 3-methoxycarbonyl-phenylboronic acid and 200 mL of a NaOH aqueous solution (10% *w*/*w*) were added and heated for 30 min. In order to adjust the pH to 1, H_2_SO_4_ was added to the resulting reaction, maintaining the solution in an ice-water bath to avoid an exothermic reaction. The resulting precipitate (solution was maintained at 4 °C overnight) was filtered, rinsed three times with water and finally dried at room temperature. The corresponding synthesis reaction in shown in Figure 1.

### 2.2. 5-Terbuthyl-m-terphenyl-3,3″-dicarboxylic Acid, tBTmDA

**Figure 2:**
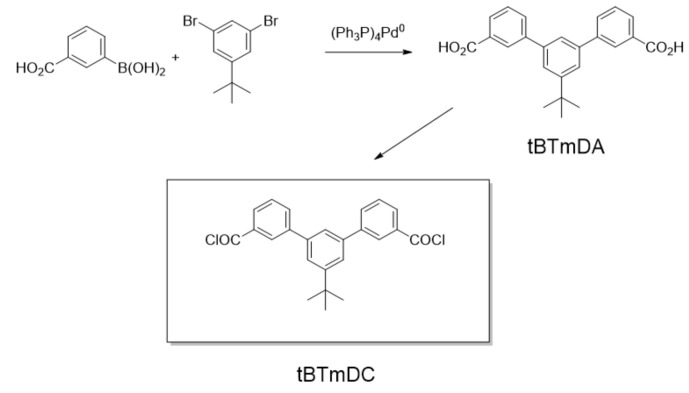
Synthesis of *m*-terphenyl-3,3″ dicarboxylic acid, tBTmDA and *m*-terphenyl-3,3″ dicarboxylic acid (tBTmDC).

The Susuki-Miyakura [29] reaction was used to obtain *m*-terphenyl-3,3″-dicarboxylic acid, which involved C-C coupling through a reaction of a boronic derivative (R^1^-B(OH)_2_) and an organohalogenide (R^2^-X) in the presence of a base and a Pd(0) catalyst (schematized in Figure 2).

The synthesis was carried out according to a procedure adapted from Liao and Hsieh [30], as described by Soto et al. [31]. In a round-bottomed flask, 5 g of 3-carboxy-phenylboronic acid (as coupling C-C), 3.639 g of 1,3-dibromo-5-terbuthylbencene (as the corresponding derivative), 1.362 g of Pd(PPh_3_)_4_ (as the catalyst) and 360 mL of deoxygenated anhydrous DMF were added. In order to avoid the degradation of the catalyzer, the reaction was blanketed by an inert atmosphere, before adding 79.8 mL of a deoxygenated potassium carbonate solution (3.2 M) and maintained at 85 °C for 8 h under magnetic stirring. The reaction was cooled before adding hydrochloric acid 0.1 M to obtain a precipitate at pH 1. The solid diacid was obtained through filtration and dissolved in 35 mL of NaOH (2 M). HCl (50:50) was added to adjust the precipitate pH to 1 to obtain a solid precipitate, which was filtered again, rigorously washed with water and finally dried before rinsing with warm toluene. The resulting diacid (tBTmDA) was dried at room temperature under vacuum for 24 h.

### 2.3. Synthesis of Diacid Chloride

The synthesis of the dichloride acid, tBTmCl, was carried out by using thionyl chloride [32].

Thus, in a round-bottomed flask equipped with a reflux condenser and whilst being magnetically stirred, SOCl_2_ and 5 drops of DMF were added onto the diacid compound (14 g) at room temperature; afterwards, the reaction suspension was heated up to 50 °C and maintained at that temperature for 4 h and, finally, at 80 °C for 2 h.

Distillation equipment was employed to eliminate the SOCl_2_ by adding a small amount of anhydrous toluene prior to the distillation. The process was initially carried out under vacuum at room temperature. To assure that SOCl_2_ was eliminated, additional anhydrous toluene was again added to the reaction and the temperature was increased to 70 °C until the distillation was finished. The solid residue was collected, recrystallized in 20 mL of anhydrous hexane and then cooled at 4 °C to obtain a pure monomer. The recrystallized monomer tBTmCl was rinsed with hexane and dried under vacuum.

### 2.4. Polymer Synthesis

Polyamide (tBTmCl-APAF) was synthesized from the acid dichloride (tBTmCl) and the diamine (APAF) using the in situ silylation activated polyimidization method, according to the procedure reported by Muñoz et al. [33].

Polymer synthesis was carried out according to Smith et al. [32] adapted by Soto et al. [34] as follows: In a three-necked flask, equipped with a mechanical stirrer under a constant N_2_ supply, the diamine (APAF) (6.8 mmol) was added and dissolved with anhydrous DMAc (14 mL) at room temperature. Once dissolved, the mixture was cooled to 0 °C before the slow addition of CTMS (3.4 mL, 27.34 mmol), followed by pyridine (2.2 mL, 27.31 mmol). To ensure the formation of the silylated diamine, the diamine solution was raised to room temperature and allowed to stir for 10 min. The solution was again cooled to 0 °C. Subsequently, tBTmCl (6.8 mmol) was added and DMAp (0.1668 g), followed by 5 mL of DMAc. The reaction was stirred for 24 h at room temperature to complete the polymerization reaction. Polymer fibers were obtained through precipitation in water and washed twice with a 50:50 ethanol-water solution. Finally, a drying protocol was followed until solvents were completely eliminated.

### 2.5. Polymer Characterization

Weight-average molecular weight (Mw) and number-average molecular weight (Mn) of the synthesized o-hydroxypolyamide (HPA) (tBTmCl-APAF) were determined using gel permeation chromatography (GPC) using a Tosoh Ecosec HLC-8320GPC (Tosoh, Tokyo, Japan) device. The sample was prepared by dissolving 0.5 mg of polymer in 2 mL of THF and filtering through a 0.45 µm filter. Table 1 shows the molecular weight obtained with this technique, along with its polydispersity value (IP).

**Figure 3:**
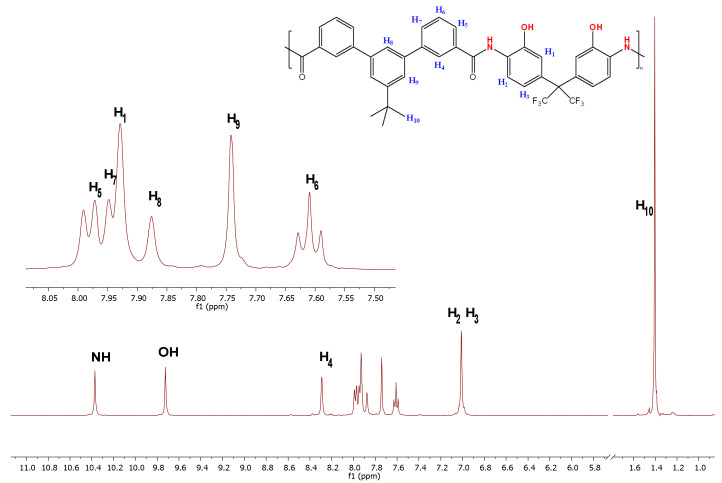
NMR spectrum of tBTmCl-APAF HPA.

The ^1^H nuclear magnetic resonance (NMR) spectra were performed using a Varian AV Agilent (Varian, Palo Alto, CA, USA) working at 400 MHz and 100 MHz, respectively. The samples were prepared using deuterated dimethyl sulfoxide (DMSO-*d6*) to dissolve the polymer. The obtained NMR spectrum for the pure tBTmCl-APAF HPA is shown in Figure 3.

Polymer solubility was determined by placing ~10 mg of the polymer in solubility tubes and adding 1 mL of the target solvent (DMAc, NMP, THF, CHCl_3_, *m*-cresol, acetone, ethanol, DMF) until its total dilution. The resulting HPA polymer turned out to be soluble in a common organic solvent such as THF and also in polar aprotic solvents such as DMAc, NMP and DMF.

### 2.6. Polymer Matrix Films

To prepare the membranes, the synthesized polymers were separately dissolved in THF 10% (*w*/*v*) and maintained under mechanical stirring until its complete dissolution. Before casting onto a glass plate, the solution was filtered through a 4.5 µm PTFE membrane syringe filter to remove impurities. After casting, part of the solvent was stripped off at room temperature overnight. The remaining solvent was slowly dried in a vacuum oven (Thermo Fisher Scientific Inc. Waltham, MA, USA) with the following thermal protocol: 60 °C for 2 h and 80 °C for 2 h without vacuum, 100 °C for 2 h, 120 °C for 1 h and, finally, 180 °C for 12 h under vacuum. The obtained membranes presented a thickness of 40–60 µm.

### 2.7. Preparation of Mixed Matrix Membranes

**Figure 4:**
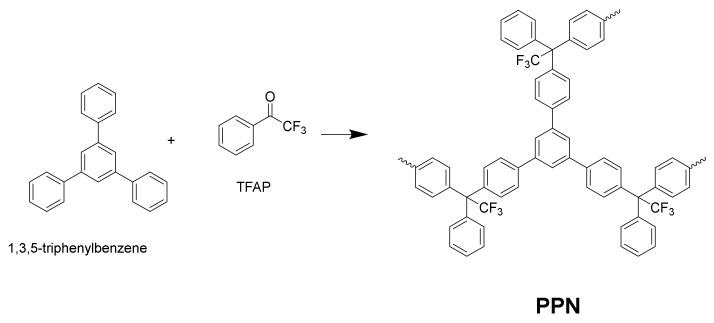
A schematic representation of the structure of the PPN synthetized from 1,3,5-triphenylbenzene and trifluoroacetophenone (TFAP) by following the procedure of Lopez-Iglesias et al. [25].

A PPN was synthetized according to Lopez-Iglesias et al. [25] from a reaction of triptycene and trifluoroacetophenone (TFAP). A sketch of its structure is shown in Figure 4.

Mixed matrix membranes (MMMs) were prepared as described elsewhere [18,31], using a solution casting method. The polymeric matrix synthesized was mixed with the porous polymer network (PPN) used as the filler.

The general procedure was as follows: 1.2 g of polymer matrix was dissolved in 10% of THF (*w*/*v*). Simultaneously, a determined amount of the filler (20 and 30% (*w*/*w*)) was dispersed in 10% (*w*/*v*) of THF. To obtain a better particle dispersion, the filler was sonicated for 20 min at 30% of maximum amplitude (40 cycles of 20 s sonication followed by 10 s cooling-down) before being mixed with the polymer matrix. The mixture was cast onto a glass casting plate and subjected to a thermal treatment under vacuum conditions to remove the solvent: 100 °C for 1 h, 120 °C for 1 h, 180 °C overnight. For comparative purposes, a neat membrane was manufactured using the protocol described above. The membrane’s thickness range was 50–60 μm.

### 2.8. Thermal Rearrangement Mixed Matrix Membranes (TR-MMMs)

**Figure 5:**
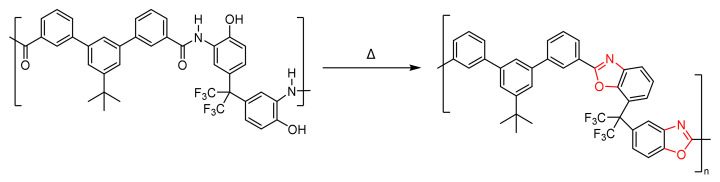
Thermal process for the conversion of *o*-hydroxypolyamide to polybenzoxazole.

The obtained membranes (HPA) MMM20 (20 w% filler) and MMM30 (30 w% filler), were cut in round pieces of approximately 3 cm^2^ and placed between two quartz plates separated by a stainless-steel washer. The thermal rearrangement of films was carried out in a Carbolite Split-Tube Furnace, under a N_2_ purge to maintain an inert atmosphere during the process. A thermal rearrangement was performed by heating the samples at a ramp rate of 5 °C/min up to 250 °C, maintained for 15 min, then increasing the temperature to 5 °C/min up to the target temperature (375 °C), and keeping the samples isothermally at that temperature for 15 min to ensure the complete conversion of the *o*-hydroxypolyamide to polybenzoxazole (Figure 5). The resulting films, TR-MMM20 (20 w% filler) and TR-MMM30 (30 w% filler) were cooled to room temperature at 10 °C/min, maintaining the inert atmosphere. The conversion was verified using FTIR as seen further below.

## 3. Membrane Characterization

### 3.1. Thermogravimetric Analysis

The thermogravimetric analysis (TGA) of the membranes was performed by using a TA Instruments Q500 thermogravimetric analyzer (TA Instruments, New Castle, DE, USA) in 5 mg samples. Ultra-high purity nitrogen at a flow rate of 40 mL/min was used. The Hi-RES method was employed setting the temperature ramp 10 °C/min up to 800 °C.

**Figure 6:**
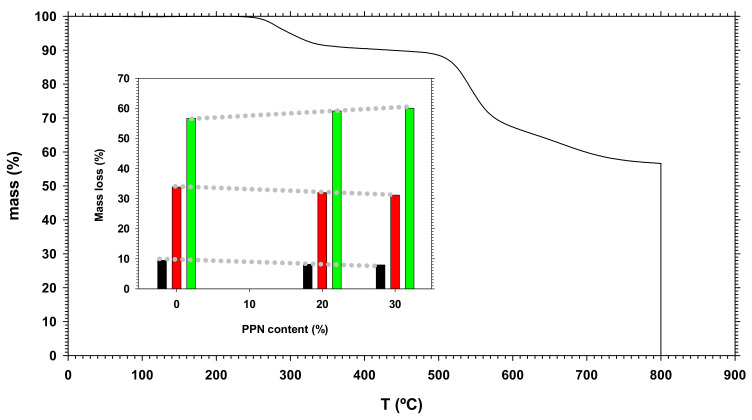
Thermogram for the pure HPA-based matrix membrane (tBTmCl-APAF) with an inset showing the three successive mass losses for the pure HPA (tBTmCl-APAF) matrix, the 20% PPN MMMs and the 30% PPN one. At 800 °C, the gas was changed from N_2_ to purified air.

The thermogram for the pure HPA-based matrix membrane (tBTmCl-APAF) is shown in Figure 6. The three successive mass losses for the pure HPA and the films with 20% PPN MMMs and 30% PPN are also shown in an inset. All weight losses happened at the same temperature for all the MMMs, no matter the employed PPN load value. The first weight loss was seen at approximately 280 °C, which can be linked to cyclodehydration (the conversion of the HPA to benzoxazole) along with some amount of trapped solvent (weight loss values between 7.9 and 9.5%). The second step (at approximately 540 °C) is associated with the generalized thermal degradation of the polymer matrix and the PPN structure [35,36,37,38]. The weight residue at 800 °C (under N_2_) corresponded to the char yield. After swapping the N_2_ atmosphere to an air one, the final residue was zero. In the graph, we can see the corresponding mass losses that seemed to decrease with increasing PPN contents.

### 3.2. Differential Scanning Calorimetry

**Figure 7:**
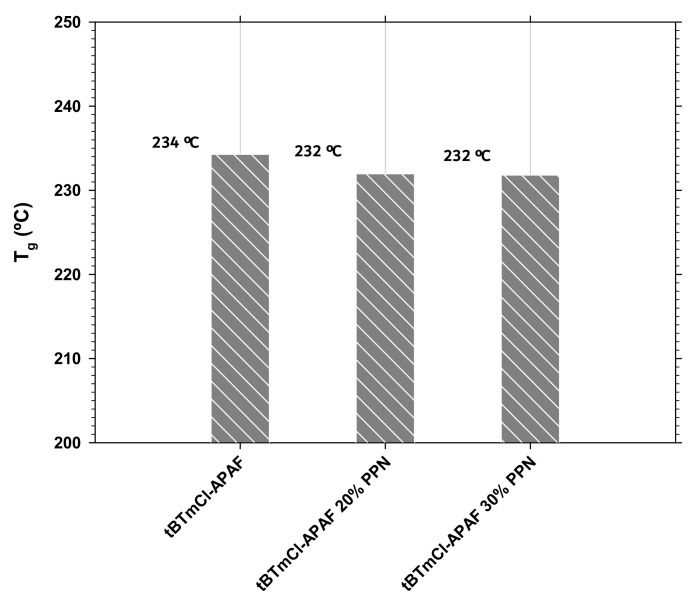
Glass transition temperatures for tBTmCl-APAF with different PPN loads.

To monitor the glass transition temperature (Tg), differential scanning calorimetry (DSC) was carried out in a TA Instruments DSC Q-20 Analyzer (TA Instruments-Water Corp. Milford, MA, USA). DSC analyses for TR polymers were carried out at a heating rate of 20 °C/min up to 360 °C, while for non-thermally rearranged polymers, the use of modulated DSC (MDSC) was carried out at 20 °C/min up to 320 °C, under a N_2_ atmosphere for both materials using 6–10 mg of the membrane in gas-tight aluminum containers. The glass transition temperature (Tg) was determined in the second heating cycle from the middle point of the resulting Cp slopes. In Figure 7, the glass transition temperatures are shown for our membranes. Note that Tg (and rigidity) remained almost constant when the added PPN was less than 2 °C.

### 3.3. Fourier Transform Infrared Spectroscopy (ATR-FTIR)

The influence of the filler on the MMMs was studied via attenuated total reflectance-Fourier transform infrared (ATR-FTIR) spectroscopy using a Perkin Elmer Spectrum One FT-IR (Perkin-Elmer, Waltham, MA, USA) coupled with a universal diamond-tipped attenuated total reflection (ATR) sampling module.

**Figure 8:**
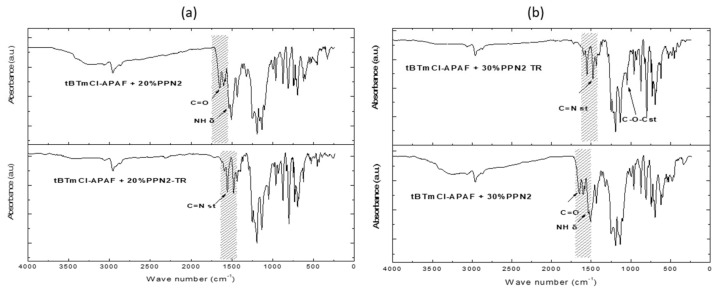
FTIR absorbance spectra for the tBTmCl-APAF 20% PPN MMM (**a**) and the tBTmCl-APAF 30% PPN MMM (**b**).

The corresponding FTIR spectra are shown in Figure 8. O-H or N-H vibration bands (at approximately 3000 cm^−1^) could be associated with some remaining water, and are related to the hydroxyl and amine groups. A stretching vibration band for C=O (1638 cm^−1^) and a N-H symmetric band (1497 cm^−1^) were also observed. A stretching vibration band for the C-F group (~1200 cm^−1^) was also observed (the presence of this band was both characteristic of the matrix and the filler) [25]. The flattening of the area at approximately 3000 cm^−1^ and the small presence of C-H bending bands at approximately 540 cm^−1^ were the main contributions of PPN to the MMM spectra.

The conversion of the precursor HPA membranes to PBOs was confirmed by the appearance of C=N (1462 cm^−1^) and C-O-C (1034 cm^−1^) stretching absorption bands, which are characteristic bands of benzoxazoles. In addition, the decrease in some characteristic bands of the amide moieties could be observed due to the rearrangement process.

### 3.4. Wide-Angle X-ray Scattering

**Figure 9:**
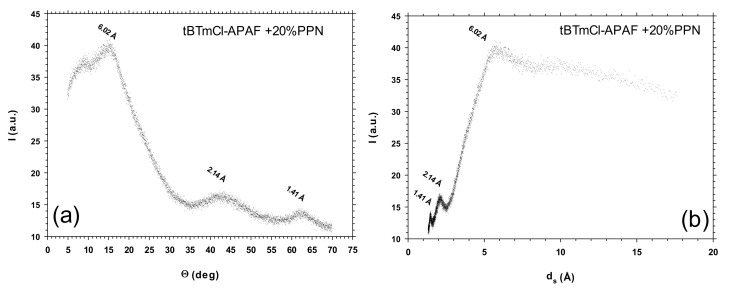
WAXS spectrum for the tBTmCl-APAF + 20% PPN membrane before thermal rearrangement in terms of the corresponding dispersion angle (**a**) and the intersegmental distance according to Bragg’s law (**b**).

The membranes were investigated via wide-angle X-ray scattering (WAXS) at room temperature using a Bruker (Bruker, Billerica, MA, USA) D8 discover A25 advanced diffractometer equipped with a *Goebel* mirror. The LynxEye detector was operated at a speed of 0.5 s with a step scanning mode ranging from 5° to 70° and a 2θ step of 0.020°. A Cu Kα (λ = 1.542 Å) radiation source in a ceramic tube was used. Figure 9 shows an example of that intersegmental chain.

Before the thermal rearrangement, the intersegmental distance increased when the load of PPN increased. After the thermal rearrangement, d_s_ was substantially constant, independent of the PPN content of the membrane. These trends are shown in Table 2.

### 3.5. Mechanicals Properties

Mechanical properties of the pure polyamides, MMMs and TR membranes were determined with a Shimadzu Autograph AGS-X 500 N tensile testing instrument (Shimadzu, Kyoto, Japan). The tensile test was performed at a crosshead speed of 1 mm/min. Samples were cut using a microtensile dogbone-shaped die before the heat treatment. The gauge length and width (~22 mm and 5 mm, respectively) were measured with a digital scanner using ImageJ software to measure the average width and gauge length. Membrane thicknesses were measured with a Mitutoyo digital caliper (Mitutoyo, Kawasaki, Kanagawa, Japan) of ±1 µm resolution. Five replicate measurements were carried out for each membrane tested.

Table 3 shows some examples on the maximum stress, elongation at break and Young’s modulus for these samples. There, it can be seen that these mechanical properties were moderate but sufficient to test these materials on gas separation applications. Note that the maximum stress decreased when the PPN percentage increased and after the thermal treatment. The elongation at break and Young’s moduli decreased when more filler was added, but increased when the thermal rearrangement was completed.

### 3.6. Gas Transport: Permeability and Selectivity

Membranes with a uniform thickness were placed on an aluminum frame (using an epoxy as the adhesive and protected with glass fiber filter paper) to determine gas permeability. This epoxy was dried at room temperature during 3 h, followed by 3 h at 60 °C before use. The dry sample was placed onto the permeation cell using the constant-volume variable-pressure permeation system [39]. Gas permeability (cm^3^ (STP) cm/(cm^2^ s cmHg)) was determined by:

(1) $$P=\frac{V_{d}\ell }{p_{2}ART}\left[{\left(\frac{dp_{1}}{dt}\right)}_{ss}-{\left(\frac{dp_{1}}{dt}\right)}_{leak}\right]$$

where $V_{d}$ is the downstream volume (cm^3^) of a permeation system, ℓ is the membrane thickness (cm), $p_{2}$ is the upstream pressure (cmHg), A is the area available for the gas transport (cm^2^), the universal gas constant R is 0.278 cmHg cm^3^/[cm^3^(STP)·K], T is absolute temperature (K) and ${\left(dp_{1}/dt\right)}_{ss}$ and ${\left(dp_{1}/dt\right)}_{leak}$ are the pressure changes (cmHg/s) in the downstream volumes when the gas permeation through the film was at the pseudo-steady state and during the leak test, respectively. Gas selectivity was:

(2) $$\alpha_{A/B}=\frac{P_{1}}{P_{2}}$$

Samples were kept under vacuum overnight at 35 °C before testing to remove any absorbed gas. The permeabilities of He, H_2_, O_2_, N_2_, CH_4_ and CO_2_ (99.999% purity) supplied by Airgas (Airgas, Radnor, PA, USA) were measured at three bars at 35 °C. He permeability was measured at one, two and three bars to detect pin holes through the membranes prior to any ulterior membrane testing. A vacuum was implemented for at least 20-fold the time lag before measuring the permeability for a new gas.

**Figure 10:**
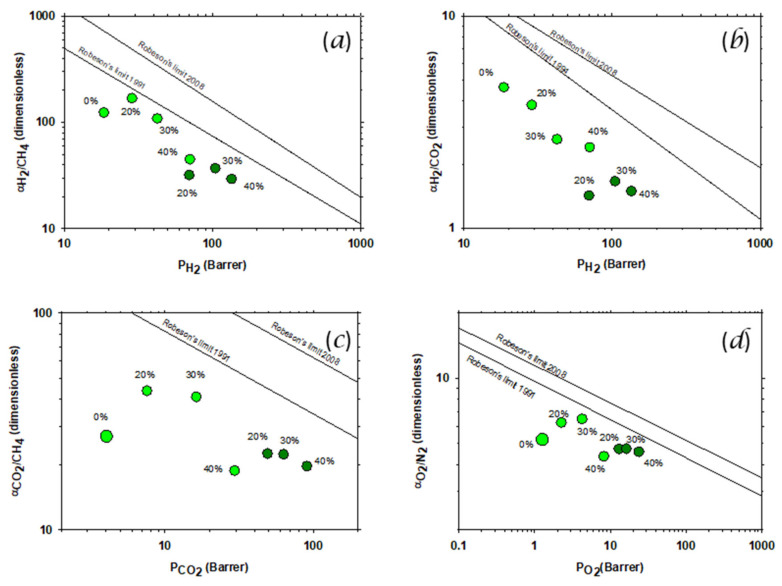
Permeability-selectivity Robeson’s plot for the H_2_/CH_4_ (**a**), H_2_/CO_2_ (**b**), CO_2_/CH_4_ (**c**) and O_2_/N_2_ (**d**) gas pairs. Darker symbols correspond to thermally rearranged MMMs with 20%, 30% and 40% PPN content. Clearer ones include also the pure HPA (tBTmCl-APAF) membrane prior to thermal rearrangement. Straight lines correspond to the 1991, 1994 and 2008 Robeson’s trade-off bonds [6,7,8].

Permeation measurements (at 35 °C and three bar) were performed to evaluate the effects of the addition of the PPN on the polymeric matrix and thermal rearrangement. The selectivity versus permeability (Robeson’s plots) for the gas pairs H_2_/CH_4_ and H_2_/CO_2_ are shown (along with corresponding trade-off lines) in Figure 10a,b. In Figure 10c,d the Robeson’s plots for CO_2_/CH_4_ (c) and O_2_/N_2_ (d) are shown. It seems clear that the addition of the PPN as a filler increased the permeability, but there was a sharper decrease (as compared to hydrogen pairs) in selectivity for high PPN contents.

It seems clear that the addition of the PPN as a filler increased the permeability, but decreased the selectivity after an initial increase (for some gas pairs) for low PPN loading. In all cases, selectivity decreased after the thermal rearrangement.

**Figure 11:**
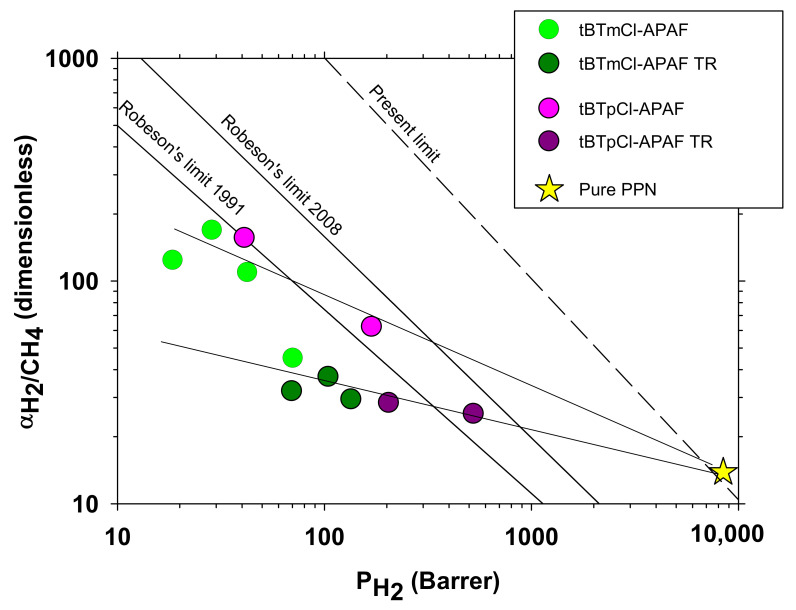
Selectivity versus permeability (Robeson’s plot) for H_2_/CH_4_ gas pair with a comparison for meta (tBTmCl APAF) and para (tBTpCl APAF). In both cases, PPN percentage increased from left to right. tBTmCl APAF MMMs include 20%, 30% and 40% PPN content. tBTpCl APAF include 0 and 20% PPN loads. The star corresponds to the pure PPN permeability [26].

These permeability versus selectivity trends were comparable, although inferior, in most cases, than those obtained previously for the isomeric tBTpCl-APAF described by Soto et al. [26], as shown in Figure 11 for H_2_/CH_4_, which could be explain by the more lineal character of the tBTmCl-APAF chain. In this figure, the corresponding extrapolated permselectivity of the pure PPN as obtained by Soto et al. [26] is also shown.

Recently [26], we proposed and tested a correlation between permeability and the fractional free volume (FFV) as:

(3) $$P\;=\;SD\;=Ae^{B\;\left(FFV\right)}$$

with:

(4) $$B\;=\;a\;+\;b\delta \;+\;c\delta^{2}$$

where δ is the kinetic diameter of the tested gas.

Combining Equations (3) and (4), we obtained:

(5) $$\ln P=\;\left[\ln A\;+\;aFFV\right]\;+\;\left[bFFV\right]\;\delta \;+\;\left[cFFV\right]\;\delta^{2}$$

**Figure 12:**
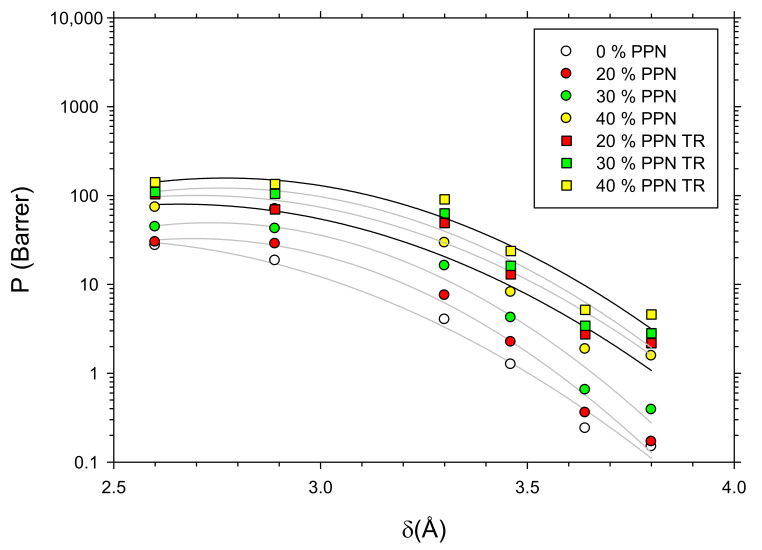
H_2_ permeability as a function of δ (gas kinetic diameters).

In Figure 12, this equation was fitted for the studied membranes, and kinetic diameters as given by Breck [40] were used. In Table 4, the parameters obtained through such a fitting procedure are shown.

### 3.7. Density and Free Volume

The calculated fractional free volume (FFV) was:

(6) $$FFV^{i}=\frac{V^{i}-V_{0}^{{}^{i}}}{V^{i}}\;i=\mathrm{HPA},\;\mathrm{PPN}$$

where $V^{i}$ is the total specific volume, while $V_{0}^{i}$ is the specific skeletal volume of the *i*-th phase (*i* = HPA; PPN). The skeletal volume for HPA and PPN can be estimated from their Van der Waals volumes as to $V_{0}^{i}\approx 1.3V_{w}^{i}$. $V_{w}^{HPA}$ and $V_{w}^{PPN}$, and, accordingly, $V_{0}^{HPA}$ and $V_{0}^{PPN}$, could be evaluated by using molecular modeling according to the Materials Studio software (BioVia Dassault Systémes, San Diego, CA, USA). The HPA-specific volume $V^{HPA}$ could be obtained from its density as $V^{HPA}=1/\rho^{HPA}$. The density was measured according to the Archimedes principle in a CP225 Analytical Balance from Sartorius (Sartorius, Göttingen, Germany) equipped with a density measurement kit. The samples were weighed in air and in high pure isooctane at room temperature. The average density from seven samples was obtained as:

(7) $$\rho^{HPA}=\rho_{C_{8}H_{18}}\frac{W_{air}}{W_{air}-W_{C_{8}H_{18}}}$$

where $\rho_{C_{8}H_{18}}$ corresponds to the isooctane’s density, $W_{air}$ to the sample weight and $W_{C_{8}H_{18}}$ stands for the weight of the sample when submerged in isooctane. Then, finally, Equation (6) allowed for the evaluation of the FFV for HPA.

The PPN specific volume could be evaluated as the sum of its skeletal specific volume $V_{0}^{PPN}$ plus the specific volume within the PPN pores $V_{p}^{PPN}$:

(8) $$V^{PPN}=V_{0}^{PPN}+V_{p}^{PPN}$$

$V_{0}^{PPN}$ was measured in an AccuPyc 1330 V2.04N (Micromeritics Instrument Corporation, Norcross, GA, USA). Here, the skeletal volume was determined by gas displacement using the volume-pressure relationship of Boyle’s law. Helium was used as the displacement medium. The sample was placed in a sealed cup of a known volume (2.5 cm^3^). Gas was introduced to the sample chamber and then expanded into a second empty chamber with a known volume. The pressure observed after filling the sample cell and the pressure discharged into the expansion chamber were measured, and then the volume was calculated. The density was determined by dividing the sample weight by the volume measured.

Furthermore, $V_{p}^{PPN}$ was measured using CO_2_ adsorption-desorption at 0 °C (273 K) in a Nova 4200 volumetric device (Quantachrome, Boynton Beach, FL, USA). Samples were degassed at 125 °C for 18 h under vacuum before the CO_2_ adsorption measurements. By again using Equations (6) and (8), the value of the PPN FFV was attained.

Finally:

(9) $$FFV_{}^{MMM}=\phi FFV_{}^{PPN}+(1-\phi )FFV_{}^{HPA}$$

This correlation between the fractional free volume and the fraction of the filler (PPN), ϕ, assumed that there was no significant interaction between the filler and matrix.

Another procedure could be followed by assuming that the Van der Waals volumes were additive:

(10) $$V_{W}^{MMM}=\phi V_{W}^{PPN}+(1-\phi )V_{W}^{HPA}$$

Allowing for the evaluation of $V_{w}^{MMM}$, from $V_{w}^{HPA}$ and $V_{w}^{PPN}$ and, correspondingly, $V_{0}^{MMM}$. Then, once $V_{0}^{HPA}$, $V_{0}^{PPN}$ and $V_{0}^{MMM}$ were known, we could obtain $V_{}^{MMM}=1/\rho^{MMM}$ and $V_{}^{HPA}=1/\rho^{HPA}$, and, finally, Equation (6) would allow for the determination of FFV. Note that this second method did not assume zero interactions by postulating additivity as in Equation (9), but only concerning the Van der Waals volumes as shown in Equation (10). Consequently, the first method here was referred to as the non-interaction method, while the second one was named here as interactive.

If we assumed that b and c parameters did not depend on the PPN content, the relative changes in *FFV* could be obtained from the values of both *b*FFV and *c*FFV of Table 5. The same results were obtained from *b*FFV as from *c*FFV, which indicated a good coherence of the procedure.

**Figure 13:**
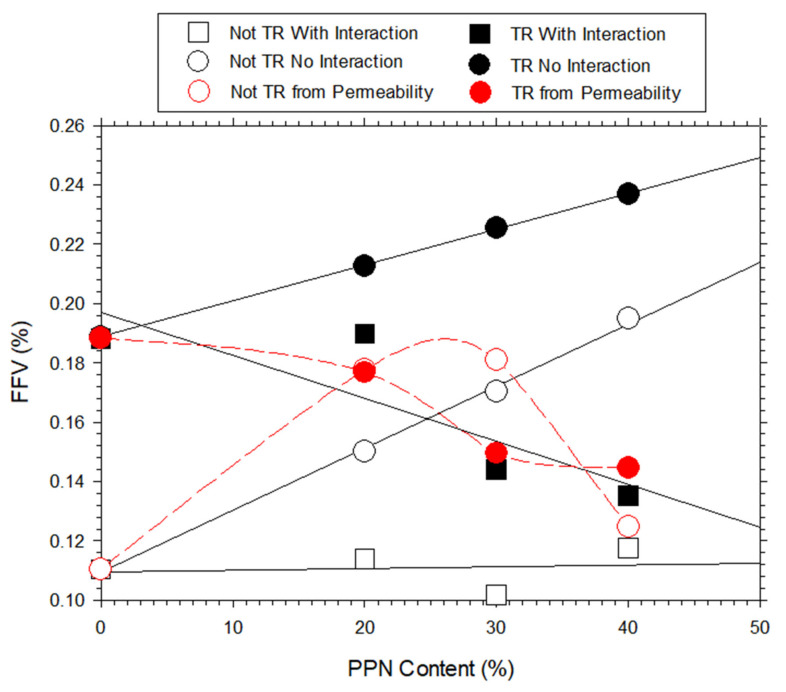
The differently calculated FFV versus PPN content.

Note that the FFV evaluated by assuming no interactions (Section 3.7) between the filler and polymeric matrix (circles in Figure 13) increased when adding the PPN both before and after the thermal rearrangement. If a matrix and a filler were supposed to interact (squares in Figure 13), the FFV decreased after the thermal treatment, while it was almost constant before the thermal rearrangement. In turn, permeability determining the FFV (red symbols in Figure 13) decreased continuously for increasing PPN loads after the thermal rearrangement, while it increased before the thermal treatment, with a steep decrease (to enter the interaction trend) for PPN contents over 30%, as shown in Figure 13. In summary, the permeable FFV for thermally rearranged membranes followed the interaction trend, while before the thermal rearrangement it followed the non-interaction trend until relatively high PPN loads were reached. This should mean that the addition of the PPN increased the trapped fractional free volume that remained inaccessible to the penetrating gases. If we took into account this fact, both Figure 10 and Figure 13 seemed to indicate that a non-interaction process between the filler and the polymer matrix corresponded to a slight increase in permeability and a high increase in selectivity. Moreover, permeability increases while selectivity decreases when the interaction between the filler and the matrix is important. This fact should be compatible with the existence of a loose interface between the filler and the polymeric matrix that would play a key role when the load is relatively high or after the thermal rearrangement process.

## 4. Conclusions

Thermally rearranged mixed matrix membranes (TR-MMMs) for gas separation were created from aydroxypolyamide (HPA) by reacting 2,2-bis(3-amino-4-hydroxy phenyl)-hexafluoropropane (APAF) with the dichloride of the 5′-terbutil-*m*-terfenilo-3,3″-dicarboxylic acid (tBTmCl) loaded with a porous polymer network (PPN) filler. The addition of increasing amounts of PPN to the polymeric matrix resulted in increased gas permeability, but decreasing selectivity. The thermal rearrangement of HPA MMMs led to the corresponding polybeonzoxazole MMMs with an additional increase in permeability, together with a decrease in selectivity.

The thermogravimetric analysis determined that the presence of the PPN filler did not lead to a decrease in thermal stability.

It was observed that the glass transition temperature of the MMs (and, hence, the inherent rigidity) increased very slightly as a function of the load employed. This loss of rigidity coexisted with an oscillation of the most likely segmental length of the polymer chain, which was almost constant after the thermal rearrangement. As for the permeability, this was slightly lower for the isomer *m*-terphenyl employed in this work when compared to the polyamide derived from the tBTpCl isomer under the same conditions, with both materials having a similar selectivity. This fact could be explained by the slightly lower stiffness of the polyamide chain derived from tBTmCl.

The intersegmental distance (ds) initially decreased and then increased as the PPN loading increased in the non-thermally treated membranes. After the thermal rearrangement, ds was substantially constant, independent of the PPN content of the membrane. This could be probably due to the rigidification of the chain caused by both the matrix-loading and the thermal rearrangement process. For all MMMs, the mechanical properties were good enough to be employed in gas separation applications. The maximum stress decreased when the PPN percentage increased, and also after the thermal treatment, although elongation at break and Young’s moduli decreased with increasing PPN contents, but both decreased when more filler was added and increased when the thermal rearrangement was accomplished.

The permeability determined by the FFV showed that the interaction between the filler and the matrix was decisive after the thermal rearrangement, and also for a relatively high PPN loading content before the thermal rearrangement. It seems reasonable to figure out that the interaction between the polymeric matrix and the PPN filler contributed to the increase in permeability while decreasing selectivity. This fact would be consistent with the apparition of a loose interface, or the presence of voids, surrounding the filler.

## Figures and Tables

**Table 1:** Molecular weight and polydispersity value of tBTmCl-APAF.

Sample	Mw (g/mol)	Mn (g/mol)	Mw/Mn
tBTmCl-APAF	104 828	58 936	1.8

**Table 2:** *d*-spacing (Å) as obtained from the maxima of WAXS spectra.

Membrane	Before TR Process	After TR Process
tBTmCl-APAF	9.93	6.29
tBTmCl-APAF + 20% PPN	6.02	6.24
tBTmCl-APAF + 30% PPN	7.11	6.10
tBTmCl-APAF + 40% PPN	10.92	6.23

**Table 3:** Mechanical properties of PA and MMMs.

Membrane	Maximum Stress (MPa)	Elongation at Break (%)	Young’s Modulus (GPa)
tBTmCl-APAF	25.7 ± 10.2	9.90 ± 1.25	1.7 ± 0.6
tBTmCl-APAF + 20% PPN	36.8 ± 6.4	2.02 ± 0.39	2.2 ± 0.2
tBTmCl-APAF + 30% PPN	31.9 ± 10.2	1.38 ± 0.39	1.7 ± 0.3
tBTmCl-APAF + 30% PPN TR	30.8 ± 7.5	1.96 ± 0.47	1.9 ± 0.1

**Table 4:** Constants in Equation (5) evaluated through fitting as shown in Figure 11.

	PPN Loading	*ln*A + *a*FFV	*b*FFV	*c*FFV
Before TR	0% PPN (r = 0.9886)	6.5 ± 1.5	6.5 ± 1.5	−1.3 ± 0.3
	20% PPN (r = 0.9760)	10.6 ± 2.8	10.6 ± 2.8	−2.0 ± 0.4
	30% PPN (r = 0.9760)	10.9 ± 4.1	10.9 ± 4.1	−2.0 ± 0.4
	40% PPN (r = 0.9760)	7.6 ± 1.8	7.6 ± 1.8	−1.4 ± 0.5
After TR	20% PPN (r = 0.9557)	−8.8 ± 2.7	10.3 ± 1.9	−2.0 ± 0.5
	30% PPN (r = 0.9251)	−10.5 ± 2.7	9.1 ± 2.0	−1.7 ± 0.3
	40% PPN (r = 0.9251)	−10.1 ± 3.4	8.9 ± 1.5	−1.6 ± 0.4

**Table 5:** FFV referred to the neat tBTmCl-APAF polyamide as obtained from the fitted parameters shown in Table 4.

	PPN Loading	FFV from *b*FFV	FFV from *c*FFV
Before TR	20% PPN (r = 0.9760)	1.63	1.60
	30% PPN (r = 0.9760)	1.68	1.60
	40% PPN (r = 0.9760)	1.17	1.08
After TF	20% PPN (r = 0.9251)	1.59	1.60
	30% PPN (r = 0.9251)	1.40	1.31
	40% PPN (r = 0.9251)	1.37	1.25
